# Hypertension as an Independent Risk Factor for In-Patient Mortality in Hospitalized COVID-19 Patients: A Multicenter Study

**DOI:** 10.7759/cureus.26741

**Published:** 2022-07-11

**Authors:** Hasan Mirza, Muhammad Atif Masood Noori, Hafsa Akbar, Hardik Fichadiya, Ikwinder Preet Kaur, Sonali Sachdeva, Jagpreet Grewal, Muhammad Zain Khakwani, Howard Levitt, Wang Chang, Najam Wasty, Chandler Patton, Ajay Shah, Priya Angi, Mohsin S Mughal

**Affiliations:** 1 Internal Medicine, Berkshire Medical Center, Pittsfield, USA; 2 Internal Medicine, Rutgers Health - Trinitas Regional Medical Center, Elizabeth, USA; 3 Internal Medicine, Jefferson Abington Hospital, Philadelphia, USA; 4 Internal Medicine, Trinitas Regional Medical Center, Elizabeth, USA; 5 Internal Medicine, Monmouth Medical Center, Long Branch, USA; 6 Medicine, Lady Hardinge Medical College, New Delhi, IND; 7 Internal Medicine, Rutgers Health - Newark Beth Israel Medical Center, Newark, USA; 8 Interventional Cardiology, Newark Beth Israel Medical Center, Newark , USA; 9 Cardiology, Newark Beth Israel Medical Center, Newark , USA; 10 Public Health, Rutgers - School of Public Health, Piscataway, USA; 11 Cardiology, Rutgers Health - Newark Beth Israel Medical Center, Newark, USA; 12 Pulmonary and Critical Care, Monmouth Medical Center, Long Branch, USA; 13 Cardiology, Monmouth Medical Center, Long Branch, USA; 14 Geriatrics, Leon Hess Cancer Center, Monmouth Medical Center, Long Branch, USA

**Keywords:** morbidity, risk factors, mortality, covid-19 infection, hypertension

## Abstract

Despite the lack of direct evidence that hypertension increases the likelihood of new infections, hypertension is known to be the most common comorbid condition in COVID-19 patients and also a major risk factor for severe COVID-19 infection. The literature review suggests that data is heterogeneous in terms of the association of hypertension with mortality. Hence, it remains a topic of interest whether hypertension is associated with COVID-19 disease severity and mortality. Herein, we perform a multicenter retrospective analysis to study hypertension as an independent risk for in-hospital mortality in hospitalized COVID-19 patients. This multicenter retrospective analysis included 515 COVID-19 patients hospitalized from March 1, 2020 to May 31, 2020. Patients were divided into two groups: hypertensive and normotensive. Demographic characteristics and laboratory data were collected, and in-hospital mortality was calculated in both groups. The overall mortality of the study population was 25.3% (130 of 514 patients) with 96 (73.8%) being hypertensive and 34 (26.2%) being normotensive (p-value of 0.01, statistically non-significant association). The mortality rate among the hypertensive was higher as compared to non-hypertensive; however, hypertensive patients were more likely to be old and have underlying comorbidities including obesity, diabetes mellitus, coronary artery disease, congestive heart failure, stroke, chronic kidney disease (CKD), chronic obstructive pulmonary disease (COPD), and cancer. Therefore, multivariable logistic regression failed to show any significant association between hypertension and COVID-19 mortality. To our knowledge, few studies have shown an association between hypertension and COVID-19 mortality after adjusting confounding variables. Our study provides further evidence that hypertension is not an independent risk factor for in-hospital mortality when adjusted for other comorbidities in hospitalized COVID-19 patients.

## Introduction

Hypertension is one of the most commonly diagnosed and treated medical conditions worldwide with a significant burden on global and national health care systems. More than one billion individuals worldwide suffer from hypertension, which affects more than 30% of the adult population. Hypertension can cause a variety of disease patterns that significantly increase the population’s mortality rate. Despite the lack of direct evidence that hypertension increases the likelihood of new infections, multiple recent clinical investigations have suggested that hypertension is a major risk factor for severe COVID-19 infection [1-3].

Previous studies suggested that COVID-19 patients with underlying medical comorbidities such as hypertension, obesity, chronic cardiac and pulmonary disease, chronic kidney disease, and cancer are at greater risk of mortality and developing severe disease [4-5]. However, hypertension is known to be the most common comorbid condition in COVID-19 patients, which is associated with worse outcomes and prognosis [6]. In one study, hypertension was the most common concomitant condition in 20,982 COVID-19 patients, accounting for 12.6%, while the overall proportion of hypertension was 39.75% in the 406 COVID-19 patients who died [7]. In a study of 140 confirmed COVID-19 patients, 30% of patients had hypertension [8]. Another study included 1099 patients with confirmed COVID-19, of whom 173 had severe disease with comorbidities of hypertension (23·7%) [9]. Similarly, data from Wuhan indicated that hypertension was the most prevalent comorbidity with reported rates ranging from 15.0 to 35.6 % [9]. Age is also an important risk factor for severe COVID-19 infection. As hypertension is more frequently seen in elderly patients, it is not of great surprise to notice hypertension in the majority of COVID-19 patients and its association with mortality.

The literature review suggests that data is heterogeneous in terms of the association of hypertension with mortality. In the past, only a few studies were performed that conducted multivariate adjustments to adjust for confounding variables and studied the association of hypertension with disease severity of COVID-19 and related mortality. Guan et al. showed a significant association between hypertension and COVID-19 severity or mortality [9]. Conversely, Huang et al. failed to show any significant association [10]. Hence, it remains a topic of interest whether hypertension is associated with COVID-19 disease severity and mortality. Herein, we perform a multicenter retrospective analysis, to study hypertension as an independent risk for in-hospital mortality in hospitalized COVID-19 patients.

## Materials and methods

Type of the study

**Figure 1:**
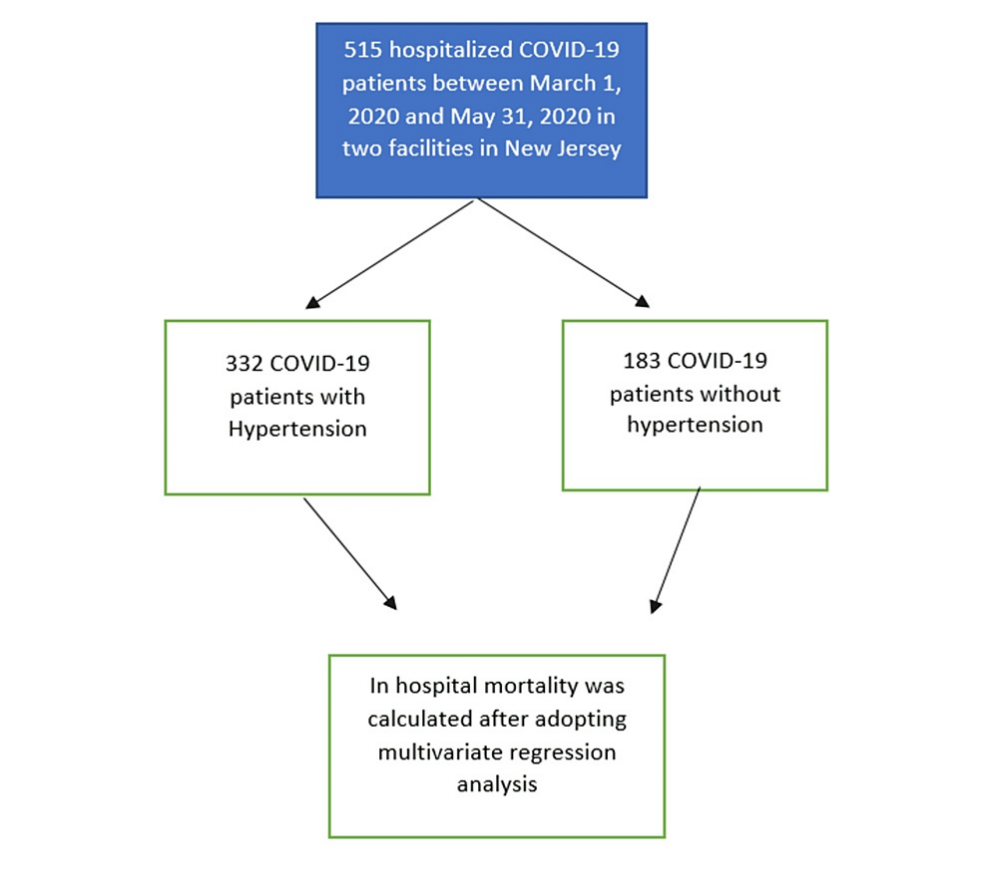
Flow Chart

The study is a multicenter, retrospective, Institutional Review Board (IRB)-approved observational study involving 515 hospitalized patients with confirmed severe acute respiratory syndrome coronavirus 2 (SARS-CoV-2) infection by nasopharyngeal polymerase chain reaction (PCR) test at Newark Beth Israel Medical Center and Monmouth Medical Center from March 1, 2020 to May 31, 2020. Permission from the respective institutional ethics committee was sought before the initiation of the study. Data were extracted manually by retrospective electronic medical records (EMR) review. The final follow-up date was May 31, 2020. Hospitalized COVID-19 patients were divided into two groups: hypertensive and normotensive. Demographic characteristics and laboratory data were collected, and in-hospital mortality was calculated in both groups (Figure 1). A multivariate regression model was adopted following a stepwise selection. Continuous variables and categorical variables were compared using the Chi-square test, Fisher’s exact, and medial two-sample test where appropriate. Statistical analysis was done with SAS software (SAS Institute, Cary, USA), and a p-value of <0.05 was considered significant.

Inclusion criteria

The study included patients with confirmed SARS-CoV-2 infection who were hospitalized for COVID-19 pneumonia. 

Exclusion criteria

The study excluded those with age <18 years and patients with COVID-19 pneumonia who did not require hospitalization.

Hospitalization criteria

Hospitalization criteria were: Patients with oxygen saturation on room air <94%, requiring oxygen therapy; patients with severe dyspnea (RR>30 breaths per minute); and patients with alteration in mental status.

Primary outcome

The primary outcomes were to determine in-hospital mortality and the association of hypertension with in-hospital mortality rates. 

Study variables

Hypertension (HTN) was considered an independent variable and in-hospital mortality was the dependent variable. The patients were divided into the hypertensive and normotensive groups. The effect of HTN on mortality was studied. Baseline demographic characteristics, comorbidities, and laboratory data were obtained.

## Results

In this indexed time period, we found 515 hospitalized COVID-19 patients, with a mean age of 63 years (49-74). The average BMI was 28.8 Kg/m2 (24.2-33.6); 256 (49.7%) were males; 201 (39.5%) were diabetics; 95 patients (18.9%) had a history of coronary artery disease (CAD); 86 patients (17.0%) had a history of congestive heart failure; 55 patients (10.9%) had a prior history of stroke; 83 patients (16.5%) had a history of chronic kidney disease (CKD); 49 patients (9.8%) had a history of chronic obstructive pulmonary disease (COPD); and 43 patients (8.6%) had a history of cancer. Of 515 patients, 256 were male and 259 were female. Among the male subgroup, 172 (51.8%), and among the female subgroup, 160 (48.2%) were hypertensives (p-value 0.19). The mean age of the hypertensive population was 68 years (57.5-77.0), while that of the normotensive population was 50 years (35.0-65.0); p-value <0.0001. The average BMI of the hypertensive population was 29.4 (24.7-34.9), while that of the normotensive group was 27.6 (23.6-32.7); p-value of 0.02. Of the 201 diabetic patients in the study, 181 (90.04%) had hypertension (p-value <0.0001). A total of 86 patients had congestive heart failure (CHF), of which 73 (84.8%) were hypertensive (p-value <0.0001). We found that 50 of 55 (90.9) patients with stroke were hypertensive (p-value <0.0001). Of the 83 patients with chronic kidney disease, 73 (87.9%) were hypertensive (p-value <0.0001). Of the 44 of 49 (89.7%) patients with chronic kidney disease were hypertensive (p-value <0.0001). Of the 43 patients with cancer, 33 (76.7%) were hypertensive (p-value 0.05, statistically non-significant association with hypertension). The overall mortality of the study population was 25.3% (130 of 514 patients) with 96 (73.8%) being hypertensive and 34 (26.2%) being normotensive (p-value of 0.01, statistically non-significant association). Patients with higher age, history of diabetes mellitus (DM), CAD, CHF, prior stroke, CKD, and COPD were more likely to have hypertension (p <0.05). However, gender, BMI, and history of cancer did not have a significant association with hypertension (Tables 1-3).

**Table 1:** Summary Statistics

Parameters	All (n=)	Summary	Hypertensive (n=332)	Normotensive (n=183)	p-value
Age	515	63.0(49.0-74.0)	68.0(57.5-77.0)	50.0(35.0-65.0)	< .0001>
Gender	515				
Male	256	256(49.7)	172(51.8)	84(45.9)	0.1995
Female	259	259(50.3)	160(48.2)	99(54.1)	
Race	515				
Black or African American	296	296(57.5)	225(67.8)	71(38.8)	< .0001>
Hispanic	101	101(19.6)	50(15.1)	51(27.9)	
Other	19	19(3.7)	9(2.7)	10(5.5)	
White	99	99(19.2)	48(14.5)	51(27.9)	
Body mass index (BMI)	497	28.8(24.2-33.6)	29.4(24.7-34.9)	27.6(23.6-32.7)	0.0214
Diabetes mellitus (DM)	509	201(39.5)	181(55.5)	20(10.9)	< .0001>
Coronary artery disease (CAD)	502	95(18.9)	89(27.9)	6(3.3)	< .0001>
Congestive heart failure (CHF)	505	86(17.0)	73(22.7)	13(7.1)	< .0001>
Cerebral vascular accident (CVA)	503	55(10.9)	50(15.6)	5(2.7)	< .0001>
Chronic kidney disease	502	83(16.5)	73(22.9)	10(5.5)	< .0001>
COPD	502	49(9.8)	44(13.8)	5(2.7)	< .0001>
Cancer	501	43(8.6)	33(10.4)	10(5.5)	0.0587
Fibrinogen mg/dl	121	509.0(414.0-658.0)	493.0(369.0-658.0)	523.0(452.0-700.0)	0.4001
D-Dimer mg/L of fibrinogen equivalent units (FEU) (interquartile range)	372	1.47(0.8-3.5)	1.5(0.8-3.6)	1.4(0.7-3.1)	0.4480
Ferritin ng/mL (interquartile range)	409	789.0 (406.0-1564.0)	793.0(410.0-1581.0)	767.0(400.0-1539.0)	0.9451
ICU admission	503	139(27.6)	100(31.1)	39(21.6)	0.0221
Non-survivors	514	130(25.3)	96(28.9)	34(18.7)	0.0107

**Table 2:** Analysis of Maximum Likelihood Estimates Only age and gender are significant.

Parameter		Degrees of Freedom	Estimate	Standard Error	Wald Chi-Square	Pr > Chi-Square
Intercept		1	-3.3159	0.6033	30.2115	< .0001
Hypertension	Yes	1	-0.1363	0.2859	0.2273	0.6336
Gender	F	1	-0.6146	0.2266	7.3585	0.0067
Age		1	0.0314	0.00802	15.363	< .0001
BMI		1	0.016	0.00962	2.7672	0.0926
Diabetes mellitus	Yes	1	-0.0308	0.2506	0.0151	0.9023
Chronic obstructive pulmonary disease (COPD)	Yes	1	-0.2529	0.3863	0.4287	0.5126
Cerebral vascular accident (CVA)/transient ischemic attack (TIA)	Yes	1	0.2443	0.3418	0.5107	0.4748
Cancer	Yes	1	0.335	0.3655	0.8399	0.3594
Coronary artery disease (CAD)	Yes	1	0.1356	0.3175	0.1824	0.6693
Congestive heart failure (CHF)	Yes	1	0.3964	0.3115	1.6197	0.2031

**Table 3:** Hosmer and Lemeshow Goodness-of-Fit Test P=0.1621 >0.05. Therefore, the model is a good fit.

Chi-Square	Degrees of freedom	Pr > Chi-Square
11.7632	8	0.1621

## Discussion

In this multicenter retrospective study, we have studied the association between hypertension and in-hospital mortality in patients with COVID-19. Advanced age, hypertension, diabetes, cardiovascular disease, morbid obesity, and elevated D-dimer values have all been shown to assist clinicians in identifying patients with poor prognoses early on [11-13]. Most risk factors, rather than being independent, have a combined effect on COVID-19's negative outcomes to some level. The goal of this study was to study hypertension as an independent risk factor in the COVID-19 in-hospital mortality. Because patients with coexisting comorbidities have lower baseline well-being, it's important to compare baseline data (such as age and main comorbidities) between hypertension and non-hypertensive individuals by eliminating potential confounding variables. Our results indicated that hypertension was present in more than 60% of the patient population; however, it failed to show any significant association (independent association) with in-patient mortality.

There have been some uncertainties in previous studies regarding the association between hypertension and mortality in COVID-19 patients. Guan et al. reported that hypertension was more prevalent in that COVID-19 patients who lead to the primary endpoint (admission to an intensive care unit, the use of mechanical ventilation or death; 35.8% versus 13.7%) and in those with severe disease (23.7% versus 13.4%) [9]. A study conducted by Ruan et al. also described that patients with cardiovascular disease and hypertension had a higher mortality rate as compared to patients that were discharged [14]. Yang et al. also reported that hypertension was more prevalent in severe COVID-19 patients. However, none of the above studies performed multivariate regression, which could falsely associate hypertension with COVID-19 severity and mortality due to potential confounders. A meta-analysis by Du et al. included 24 observational studies with 99,918 COVID-19 patients and showed that hypertension was associated with a significantly increased risk of in-hospital mortality of COVID-19 [15].

In our study, performed on 515 patients with confirmed COVID-19, 332 (64.4%) patients had underlying hypertension. Hypertensive patients were more likely to be old and have underlying comorbidities including obesity, diabetes mellitus, coronary artery disease, congestive heart failure, stroke, CKD, COPD, and cancer. The mortality rate among hypertensive was higher as compared to non-hypertensive, but multivariable logistic regression failed to show any significant association between hypertension and COVID-19 mortality. Similar to our study, Huang et al. failed to show a significant association between hypertension and COVID-19 severity and mortality after conducting multivariate adjustments [10]. Another study, by Mcfarlane et al., found no significant association between hypertension and in-hospital mortality after multivariate adjustment for confounding variables [16]. 

The study was conducted only on patients hospitalized for COVID-19 pneumonia. Patients under 18 years of age were not included in the study. Hence further studies would be needed to study the effect of hypertension as an independent variable of mortality for COVID-19 patients treated in outpatient settings and for patients under 18 years of age. Also, a bigger study with a large sample would be required to validate the results of this study.

## Conclusions

This multicenter data showed that hypertension is not an independent risk factor for in-hospital mortality in hospitalized COVID-19 patients. To our knowledge, few studies have shown an association between hypertension and COVID-19 mortality after adjusting confounding variables. Our study provides further evidence that hypertension is not an independent risk factor for in-hospital mortality when adjusted for other comorbidities in hospitalized COVID-19 patients. Future high-quality randomized controlled trials (RCTs) and meta-analyses are necessary to consolidate the implications of our findings and navigate the trajectory of our understanding toward the best patient-related outcomes.
